# Introducing Plagiarism and Its Aspects to Medical Researchers is Essential

**DOI:** 10.5812/aapm.9903

**Published:** 2013-03-26

**Authors:** Mahsa Ghajarzadeh, Mehdi Mohammadifar, Saeid Safari

**Affiliations:** 1 Brain and Spinal Injury Repair Research Center (BASIR), Tehran University of Medical Sciences, Tehran, Iran; 2 Department of Anesthesiology, Rasoul Akram Medical Center, Iran University of Medical Sciences (IUMS), Tehran, Iran

**Keywords:** Plagiarism, Ethics, Journalism, Medical, Publishing

Dear Editor,

Scientific misconduct and academic dishonesty have become crucial issues in the field of medical research in recent years. They include fabrication, falsification, plagiarism and deception. plagiarism is the most common form which is derived from the Latin word “plagiare” which means “kidnap” and defined as “not permitted, misappropriation of another’s work, ideas, methods, results or words without granting the source and original instigator” (1, 2). Self-plagiarism which defines as misconduct of one’s own work is another challenging issue. There is controversy to consider it as plagiarism of other's work or not (3). Previous studies reported that rate of plagiarism among medical students was 56% in America while this rate was 90 % in Croatia (1, 4). On the other hand, Martinson et al. stated that 2% of authors applied ideas of others without any permission or acknowledging the owner (5). Different factors have been suggested to contribute to the act of plagiarisms: lack of proficiency in English in non-English speaking countries, social and academic benefits, and being not familiar with respecting intellectual properties (6). Familiarity with an issue such as plagiarism and considering its consequences will be helpful to avoid this type of academic misconduct. Our study showed that medical students of Tehran University are not familiar enough with this problem (7) while Shirazi et al. reported that medical faculty members of Pakistan University are more familiar with plagiarism than medical students (8). Identification of plagiarism becomes easier by means of different softwares. Softwares such as w-copy find can be downloaded from internet (www.plagiarism. Phys. Virginia. Edu/w software. html) which evaluates document files to find matching phrases between them. The software is free and can be applied in any languages. The other plagiarism detection program is Glatt plagiarism service which omits every fifth words to assess plagiarism. If the author could not fulfill more than 77 % of missing words, it would be identified as plagiarism (9). Introducing plagiarism and its aspects to researchers by providing materials like leaflets, brochures, encouraging them to participate in medical ethics courses or workshops along with consider warning against it, will be effective in plagiarism reduction because unfamiliarity with this issue is an important leading factor.

## References

[1] Bilic-Zulle L, Frkovic V, Turk T, Azman J, Petrovecki M (2005). Prevalence of plagiarism among medical students.. Croat Med J..

[2] Cronin SN (2007). The problem of plagiarism.. Dimens Crit Care Nurs..

[3] Mavrinac M, Brumini G, Bilic-Zulle L, Petrovecki M (2010). Construction and validation of attitudes toward plagiarism questionnaire.. Croat Med J..

[4] Rennie SC, Crosby JR (2001). Are "tomorrow's doctors" honest? Questionnaire study exploring medical students' attitudes and reported behaviour on academic misconduct.. BMJ..

[5] Martinson BC, Anderson MS, de Vries R (2005). Scientists behaving badly.. Nature..

[6] Vasconcelos S, Leta J, Costa L, Pinto A, Sorenson MM (2009). Discussing plagiarism in Latin American science. Brazilian researchers begin to address an ethical issue.. EMBO Rep..

[7] Ghajarzadeh M, Hassanpour K, Fereshtehnejad SM, Jamali A, Nedjat S, Aramesh K (2012). Attitude towards plagiarism among Iranian medical students.. J Med Ethics..

[8] Shirazi B, Jafarey AM, Moazam F (2010). Plagiarism and the medical fraternity: a study of knowledge and attitudes.. J Pak Med Assoc..

[9] Austin MJ, Brown LD (1999). Internet plagiarism: Developing strategies to curb student academic dishonesty.. Internet high educ..

